# A Critical Appraisal of the Most Recent Investigations on the Hepatoprotective Action of Brazilian Plants

**DOI:** 10.3390/plants11243481

**Published:** 2022-12-12

**Authors:** Jéssica Amanda Andrade Garcia-Manieri, Vanesa Gesser Correa, Emanueli Backes, Anacharis Babeto de Sá-Nakanishi, Lívia Bracht, Jurandir Fernando Comar, Rúbia Carvalho Gomes Corrêa, Rosane Marina Peralta, Adelar Bracht

**Affiliations:** 1 Departamento de Bioquímica, Universidade Estadual de Maringá, Maringá 87020-900, Brazil; 2 Programa de Pós-Graduação em Tecnologias Limpas, Instituto Cesumar de Ciência, Tecnologia e Inovação—ICETI, Universidade Cesumar—UNICESUMAR, Maringá 87050-900, Brazil; 3 Centro de Investigação de Montanha (CIMO), Instituto Politécnico de Bragança, Campus de Santa Apolónia, 5300-253 Bragança, Portugal

**Keywords:** liver damage, co-products, bioactive compounds, antioxidants

## Abstract

Conventional treatments for liver diseases are often burdened by side effects caused by chemicals. For minimizing this problem, the search for medicines based on natural products has increased. The objective of this review was to collect data on the potential hepatoprotective activity of plants of the Brazilian native flora. Special attention was given to the modes of extraction, activity indicators, and identification of the active compounds. The databases were *Science direct*, *Pubmed*, and *Google Academic*. Inclusion criteria were: (a) plants native to Brazil; (b) studies carried out during the last 15 years; (c) high-quality research. A fair number of communications met these criteria. Various parts of plants can be used, e.g., fruit peels, seeds, stem barks, and leaves. An outstanding characteristic of the active extracts is that they were mostly obtained from plant parts with low commercial potential, i.e., by-products or bio-residues. The hepatoprotective activities are exerted by constituents such as flavonoids, phenolic acids, vitamin C, phytosterols, and fructose poly- and oligosaccharides. Several Brazilian plants present excellent perspectives for the obtainment of hepatoprotective formulations. Very important is the economical perspective for the rural producers which may eventually increase their revenue by selling increasingly valued raw materials which otherwise would be wasted.

## 1. Introduction

The importance of the liver for an organism can hardly be overemphasized as it exerts many biosynthetic and degradative functions in addition to storage, secretion, and detoxification activities. Being located between the systemic and portal circulation, the liver is continuously exposed to a variety of chemical compounds, many of them toxins and drugs, that can damage its cells (hepatocytes). The liver is no doubt the main site of biotransformation and detoxification and the latter function, especially when overwhelmed, becomes very often a serious threat to public health [1].

It has been estimated that around 10% of the world population might be affected by some kind of hepatic illness. Among these are hepatitis B and C, alcoholic liver disease, non-alcoholic fatty liver disease, hepatic cirrhosis, hepatic insufficiency, and hepato-cellular carcinoma [2,3]. One of the most notable medicines presently in use for hepatoprotection is the flavolignan fraction (generally known as silymarin) extracted from *Silybum marianum* [4,5]. In spite of its relative efficiency, there is still a widespread search for medicines capable of protecting the liver against damage in a highly efficient way, especially for compounds that are capable of regenerating damaged hepatic cells. The search for preparations derived from plants is largely based on the general belief that medicinal preparations from plants present low toxicity and that a patient’s acceptance is more favorable. The latter inserts into the current trend in health and nutrition which reflects the desire of consumers for natural plant-based products [6,7]. Plant-based therapy is frequently justified by the presence of bioactives such as phenols, flavonoids, and polysaccharides, all compounds for which hepatoprotective properties have been claimed [8].

There are many reports about the hepatoprotective abilities of extracts, infusions, and other kinds of preparations from Brazilian native plants [9]. Not all reports conform with more rigorous scientific criteria, as a significant fraction is based mainly on records of popular traditions (vox populi is the main source) or on observations that did not follow more rigorous scientific criteria [9]. The foremost purpose of this review is to perform a bibliographic survey on the most recent studies (last 15 years) on the potential hepatoprotective potential of Brazilian plants in the belief that such studies are more likely to have followed the currently acceptable standards in terms of quality and reliability of the pertinent indicators. Among the main features to be highlighted are the ways by which the raw materials were processed for producing a formulation that can be administered to experimental animals and eventually to future patients. Considering the importance of in vivo confirmation of hepatoprotective effects, the bibliographic search was strongly directed toward investigations in which animal models of hepatic lesions were used. Further, consideration was also given, whenever possible, to the bioactives that might be responsible for the observed hepatoprotective effects, especially when there are hints about mechanisms of action. The searches were done in *Science Direct, Pubmed,* and *Google Academic*, using the keywords “Brazilian native plants”, “hepatoprotective activity”, “hepatic lesion” and their variants in both English and Portuguese. The inclusion criterium was any Brazilian native plant whose hepatoprotective activity had been consistently confirmed by a fair number of widely accepted indicators. This criterion is most likely to be fulfilled by most recent studies. Plants that are amply cultivated in Brazil were not included even though, for a good number of them, well-adapted cultivars might have been developed in the country. The search dates from the first six months of the year 2022.

## 2. Hepatoxicity and the Main Toxic Agents Utilized When Studying Hepatoprotection

Hepatoprotection is mostly studied in vivo using animal models of hepatotoxicity. Hepatotoxicity is defined as a lesion in the liver associated with a compromised hepatic function, caused by exposition to medicine or to any other non-infectious agent. The hepatotoxic agents can react with basic cellular components and, consequently, induce almost all kinds of naturally occurring hepatic lesions. Drug-induced liver injury is by itself a clinical problem with an incidence from 14 to 19 cases per 100,000 persons, frequently accompanied by jaundice (30%). Furthermore, drug-induced liver injury is estimated to be the most frequent cause of acute liver failure [10,11].

Drug-induced liver injury in animal models is of the so-called direct hepatotoxicity, which opposes idiosyncratic hepatotoxicity and also indirect hepatotoxicity. Induction of direct hepatotoxicity makes use of agents that are intrinsically toxic to the liver. These agents cause predictable and dose-dependent injuries with relatively short latency periods (1–5 days). In addition to induction with hazardous chemicals, direct liver toxicity is also frequently induced with supratherapeutic doses of therapeutic drugs, such as acetaminophen, aspirin, and amiodarone. Typical manifestations are acute hepatic necrosis, serum enzyme elevations, sinusoidal obstruction, acute fatty liver, and nodular regeneration.

Idiosyncratic liver toxicity is rare and by its very nature, it is very difficult to reproduce in animal models. It is generally caused by idiosyncratic metabolic or immunologic reactions. The causative agents can be certain types of antibiotics (macrolide), cephalosporins, amoxicillin-clavulanate, etc., but its development is not dose-related. Its latency varies between days and years and manifestations are acute hepatocellular hepatitis, mixed or cholestatic hepatitis, bland cholestasis, and chronic hepatitis. Indirect hepatotoxicity is also difficult to reproduce in animal models. It is caused by the indirect action of an agent on the liver or immune system. It can be caused, for example, by antineoplastic agents, protein kinase inhibitors, monoclonal antibodies, etc. Its manifestation includes various kinds of hepatitis and fatty liver.

Among the most common agents used to induce hepatotoxicity in animals, it is worth to mention paracetamol, carbon tetrachloride, thioacetamide, furosemide, bromobenzene, and allyl alcohol [8,12]. Except for paracetamol, they all have limited clinical relevance but are all relatively easy to use. Among the six compounds just listed, paracetamol and carbon tetrachloride are by far the most frequently used in the investigations that are the object of the present review and some of their features will be described below.

**Figure 1:**
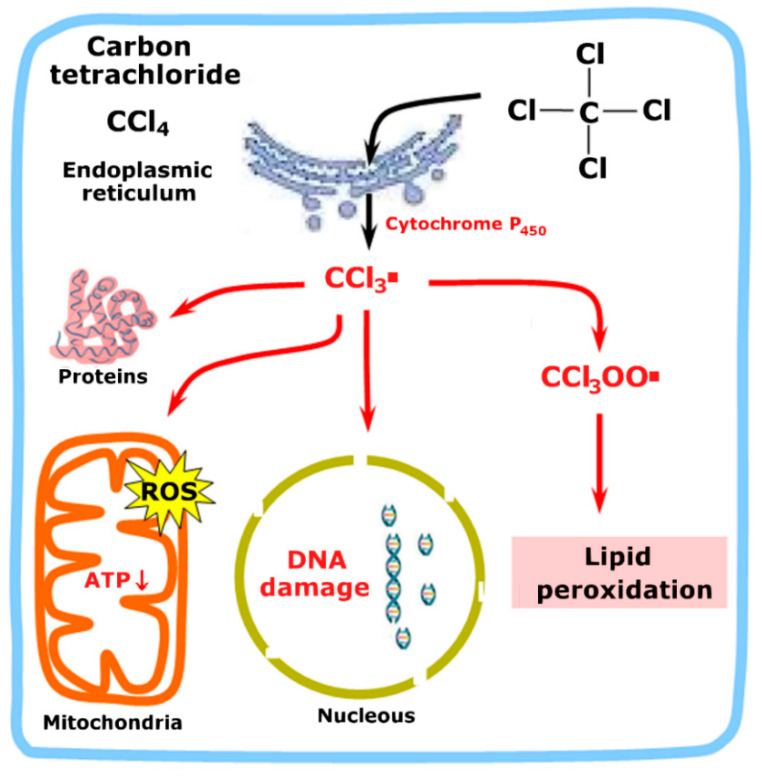
Main events of the carbon tetrachloride-induced liver injury. CCl_3_· is the trichloromethyl radical while CCl_3_OO· is the trichloromethylperoxy radical. A more detailed description can be found in the text.

The carbon tetrachloride model of liver toxicity is the most frequently used. Carbon tetrachloride (CCl_4_) is a xenobiotic that presents hepatotoxicity in humans and animals. It is found in the environment, especially in residual waters of the manufacturing sector of chlorofluorocarbons. It can also be found in cleaning fluids and fire extinguishers. As illustrated by Figure 1, the compound is initially transformed in the liver by the cytochrome P450 complex of the endoplasmic reticulum generating the reactive metabolite trichloromethyl radical (CCl_3_·) [8,12,13]. The latter binds to proteins, DNA, and lipids by means of alkylation reactions, phenomena that cause mitochondrial damage and oxidative stress, reduced protein synthesis, steatosis, and modified calcium homeostasis. Reaction with lipids destabilizes the cell membranes, which can lead to the loss of proteins. CCl_3_· also reacts with O_2_ to form CCl_3_OO· (trichloro methyl peroxy radical), which starts the lipid peroxidation chain reaction that can cause additional damage to the cellular membranes. There are indications that both major reactions, alkylation, and lipid peroxidation are required for the hepatotoxicity of carbon tetrachloride [12].

**Figure 2:**
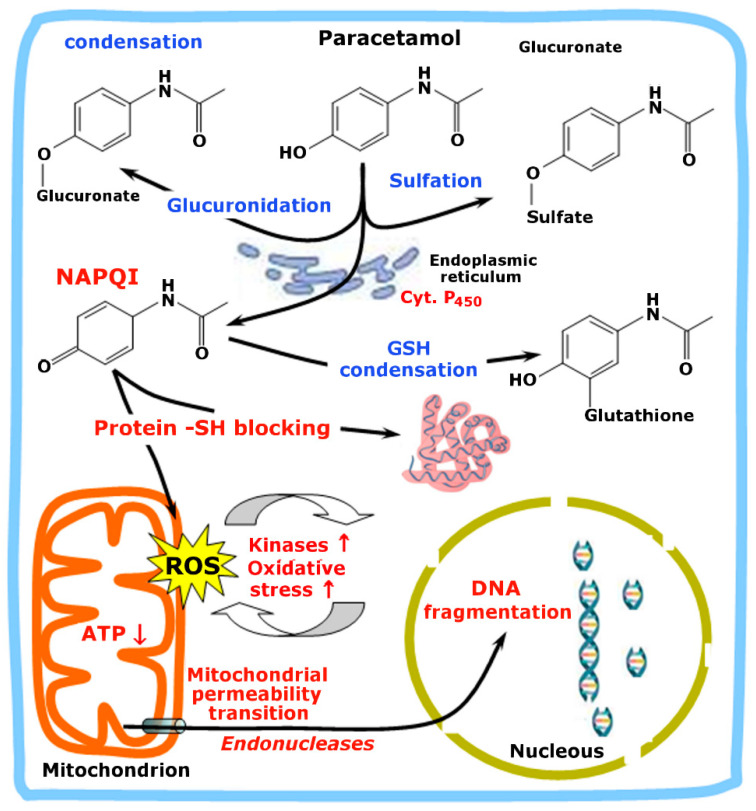
Main events of the paracetamol-induced liver injury. Upward arrows mean increase; downward ones mean decrease. NAPQI means N-acetyl-p-benzoquinone imine. A more detailed description can be found in the text.

Paracetamol or acetaminophen is a freely available analgesic. Overdosage, however, can cause serious hepatotoxicity which can lead to hepatic failure. At therapeutic doses, paracetamol is rapidly metabolized in the liver mainly through glucuronidation and sulfation reactions and just a small fraction is oxidized via cytochrome P450 into the intermediate N-acetyl-p-benzoquinone imine (NPAQI), which is highly reactive and cytotoxic (Figure 2). This compound reacts with proteins and nucleic acids resulting in widespread hepatocyte damage and death, leading to acute liver necrosis [14,15]. With low doses, this phenomenon is prevented because glutathione reacts with NPAQI, thus effectively neutralizing its deleterious effects. In case of an overdose, however, the sulfate and glucuronide pathways become saturated, and more paracetamol is shunted to the cytochrome P450 system to produce NAPQI. This event, in turn, may cause GSH depletion which further enhances the reactions of NAPQI with macromolecules. In animal studies, the liver’s stores of glutathione must be depleted to less than 70% of normal levels before liver toxicity occurs [16]. As illustrated by Figure 2, the binding of NAPQI to mitochondrial proteins causes mitochondrial dysfunction and oxidative stress. After the initial reactive oxygen species are formed, they activate kinases that further increase oxidative stress. This may eventually lead to the permeability transition, which is followed by losses of membrane polarization and ATP production. The outer membrane rupture, which occurs as a consequence of swelling, releases endonucleases into the cytosol. The translocation of these enzymes to the nucleus results in DNA fragmentation [12].

In addition to the hepatic injury models detailed above, other less common ones were used in the investigations on the hepatoprotective activity of the Brazilian plant extracts that will be described in this review. Hepatic injury also occurs, for example, as a consequence of several diseases, such as diabetes, sepsis, hyperlipidemia, and in the case of excessive alcohol intake [17].

## 3. Methods for Evaluating Hepatotoxicity

The chemical hepatotoxic agents cause molecular and structural damage to the hepatic parenchyma and modify biochemical functions. The latter can be detected as changes in the levels of several substances. Such changes can, thus, be regarded as injury markers. As a consequence of the natural impulse in neutralizing the reactive species that are produced in the experimental models of hepatic injury, a notable diminution in the antioxidant capacity of the hepatic cells takes place. The activities of superoxide dismutase (SOD), glutathione peroxidase (GPx), and catalase (CAT) are generally reduced. The same happens with the levels of reduced glutathione (GSH) in parallel with a diminution of the reduced glutathione to oxidized glutathione ratio (GSH/GSSG) [18,19]. Assay of the various antioxidant enzymes is, thus, highly revealing with respect to modifications in the oxidative status. Still, due to oxidative stress and depletion of the antioxidant defenses, the formation of secondary oxidation products, such as malonaldehyde, is equally possible. In this way, the detection of thiobarbituric acid reactive substances (TBARS assay) is generally utilized as an indicator of lipid peroxidation in the hepatic tissue [18].

Another marker of hepatic injury is the activity of the enzyme γ-glutamyltransferase (GGT), which is related to fatty liver disease induced by both alcohol and drugs [8]. Additionally, the hepatic injury reflects very strongly and in a negative way on the absorption, conjugation, and excretion of bilirubin. The increase in the plasma levels of this compound can, thus, be regarded as another marker of hepatic lesions [1].

Tissue damage generally leads to the release of enzymes into the systemic circulation. For the liver, good indicators are lactate dehydrogenase (LDH), alkaline phosphatase (ALP), alanine transaminase (ALT), and aspartate transaminase (AST) [1]. The cellular activity of all these enzymes is considerably high and their appearance in the circulation occurs as a consequence of both necrosis and increases in the cell membrane permeability [8,18]. Additionally, during hepatic injury, there is a notable alteration in blood markers such as the levels of cholesterol (total and fractions), triglycerides, creatinine, and urea [18]. Changes in protein levels in the blood have also been reported to occur as a consequence of hepatic injury, for instance, total proteins and albumin [8].

Microscopic histological observations are also often utilized for evaluating hepatic damage. A liver sample preparation may reveal dilation of blood vessels, karyolysis, lipid droplets, infiltrated inflammatory cells, and spots of necrosis [1,20].

In a less common fashion, it is also possible to determine alterations in the levels of inflammatory mediators and in the associated gene expression. During inflammatory events, activation of signaling pathways that induce the release of chemokines and cytokines in the lesioned sites occurs. In hepatic injury models, increases in the expression of interleukin-1β (IL-1 β), interleukin -8 (IL-8), inducible nitric oxide synthase, cyclooxygenase 2, and tumor necrosis factor-alpha (TNF-α) commonly occur. Moreover, the acute phase proteins alpha-1 glycoprotein and alpha-2-macroglobulin present high and low expression, respectively, in cases of hepatic lesions [21].

## 4. Extract Preparation and Characterization Procedures

The extraction of bioactive compounds from plant matrices is a prerequisite for the obtainment of effective phytotherapeutic preparations. As presented in Table 1, the bioactive compounds can be extracted from fresh, frozen, or dried plant parts. It is becoming increasingly common to encourage research on bioactives from plant parts that are not commonly used for human consumption as food. This search occurs mainly with the purpose of discovering new properties and compounds and is also determined by the current appeal for the integral use of natural products, which is in accordance with the current trend of sustainability.

With respect to the technologies that were employed in the studies discussed herein, the samples were dried basically by two methods: air drying or lyophilization. The latter is more expensive for the industrial sector but more efficient in preserving phenolic compounds as well as other compounds [22,23]. There is a relatively great number of solvents that can and that were in fact used. Most extracts were prepared using organic solvents of high efficiency and ample applicability. Methanol, ethanol, acetone, ethyl acetate, and their combinations were used for extracting phenolic compounds, frequently in combination with various proportions of water, provided that miscibility is given. The choice of solvent affects the quantity of the polyphenols that are extracted, and methanol is considered the most efficient for extracting polyphenols. Flavonoids with more than one core unit, on the other hand, are preferably extracted with aqueous acetone. However, in accordance with the principles of safety and sustainability, ethanol should be preferred if the preparation is destined for human ingestion. In addition to the organic solvents, water has also been used as an extraction medium, not only because it is cheaper, but also in accordance with the current trend toward the reduction of the use of organic solvents, which are potential environmental polluting agents [23,24].

As expected, extraction is influenced by temperature and time. An increase in the temperature of extraction can promote enhanced solubility of the analyte and increased mass transfer rates. Moreover, the viscosity and superficial tension of the solvents decrease with temperature, which helps in reaching the matrices of the sample with a consequently improved extraction rate. However, long extraction times and high temperatures increase the chances of oxidation of the phenolics, thus diminishing the functionality of such compounds. It is thus important to optimize the extraction procedure [23,25].

Besides the simple extraction by agitation, the traditional extraction methods include heat reflux and Soxhlet, boiling, lixiviation, and distillation. However, these methods have disadvantages such as the use of high temperatures (50–90 °C), which can cause thermal degradation. The methods may also imply the use of great amounts of solvents and long times of extraction [26]. Simple extraction by agitation can evidently be done at low temperatures, but in general, this diminishes the extraction efficiency. Due to these disadvantages, new methods of extraction have been developed such as ultrasound, supercritical fluids, and high hydrostatic pressure. These “green” technologies allow the use of moderate or even room temperatures, which helps to preserve thermo-sensible structures in addition to a reduction in both solvent volume and extraction time [27].

Identification and characterization of the molecular components is the final, but very important step in the study of any extract with biological activity. Presently, the most usual procedure consists of a combination of chromatographic techniques coupled to equipment that allows structure determination. High-performance liquid chroma-tography-mass spectrometry (HPLC-MS), gas chromatography-mass spectrometry (GC-MS), and more sophisticated methods such as ultra-high-performance liquid chromatography-electrospray ionization-tandem mass spectrometry (UHPLC–ESI–MS/MS) are frequently used [28,29,30,31,32,33,34,35,36]. In those cases, in which no preliminary identification is possible by means of standard samples, the investigator strongly relies on these physical methods for identification and structure determination.

## 5. Brazilian Native Plants with Potential Hepatoprotective Action Preparation and Characterization

### 5.1. General Aspects

The Brazilian plant world is considered one of the richest on our planet due to several factors: great territorial extension, climate diversity, and the existence of several biomes [37]. Brazil hosts more than 50,000 species of flowering plants, among native and non-native, and around 5000 are edible. However, only a few dozen constitute the basis of the Brazilian diet. During the last years, there has been increasing interest in finding practical uses for the numerous Brazilian native species [37].

**Figure 3:**
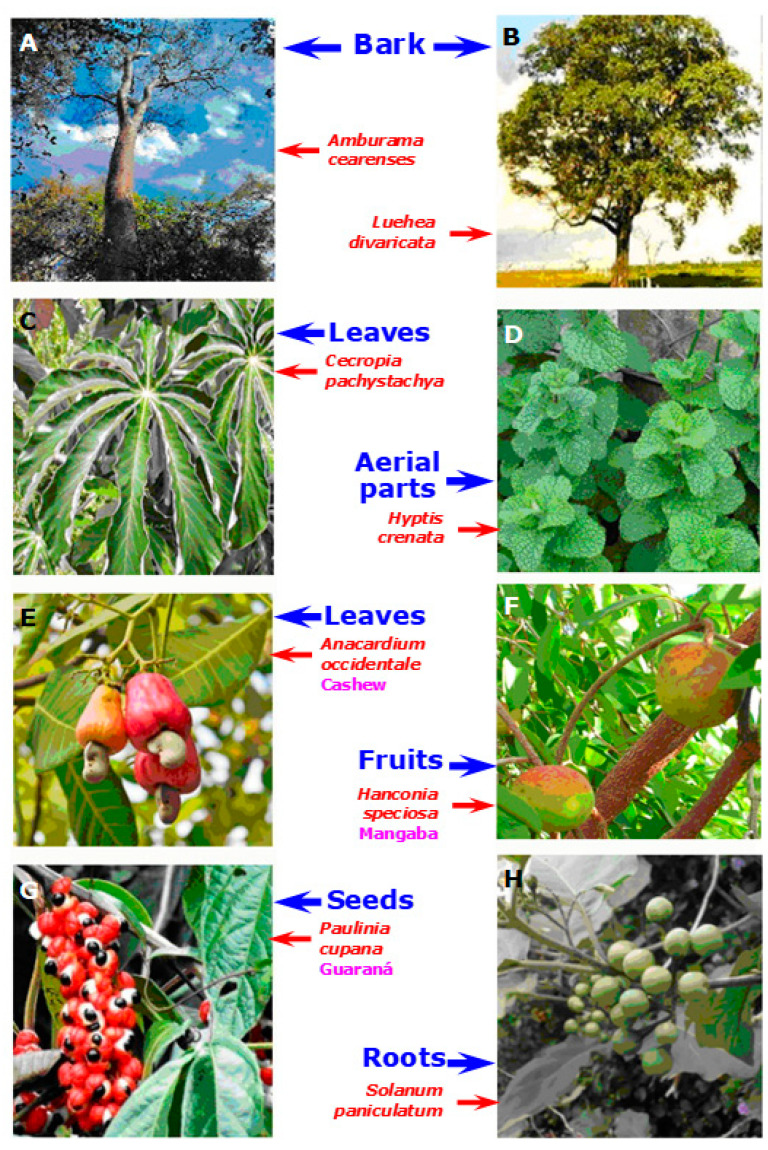
A selected group of the hepatoprotective plants described in the text and in Table 1, highlighting the parts from which the active extracts were prepared.

Table 1 shows a list of Brazilian native plants for which significant hepatoprotective activity has been demonstrated, overwhelmingly in animal models. The species are depicted in alphabetical order according to their scientific names, which precede the popular designation. A total of 26 studies were found which conform to the inclusion criteria that were established in terms of quality of the data and time period. These 26 studies refer to 22 different species. Table 1 also gives a short description of the scientific evidence about their hepatoprotective action as well as the type of extract that was used and the model of injury induction. The doses that were given to the animals are informed as well as the most important parameters that were measured. Finally, mention is made whenever possible about the hypothetical compounds that could be responsible for the reported hepatoprotective effects. Figure 3 shows photos of a selected group of plants that are listed in Table 1. This panel illustrates the ample range of plant parts that were used to prepare hepatoprotective extracts, i.e., tree barks, total aerial parts, leaves, fruits, seeds, and even roots.

### 5.2. Characteristics of the Plants with Hepatoprotective Activity and Effectiveness

Table 1 reveals that various parts of the plants can be used for obtaining preparations with hepatoprotective activity, ranging from fruit peels to stem barks. Treatment with the stem bark of *Amburana cearenses*, for example, was able to promote improvements in the pattern of liver tissue lesions, perceived by histological analyses that demonstrated reduced necrosis and diminished infiltration of inflammatory cells. In addition, parameters such as levels of hepatic enzymes and oxidative stress markers were preserved [38]. In the same way, the stem bark and the seeds of *Annona crassiflora*, normally discarded after the traditional consumption of the fruit, were active in reducing hepatic injury [39]. 

**Table 1:** Extracts from Brazilian native plants for which there is substantial evidence of hepatoprotective action. An arrow (→) preceding a solvent name is used to indicate that the first extraction was complemented by a second partition.

Scientific Name (Family)/Popular Designation/ Brazilian Biome	Plant Part/Extraction Solvent or Mode	Doses and Main Compounds	Assay; Injury Inducer	Main Results Obtained	Ref.
*Amburana cearenses* (Figure 3A; Fabaceae)/ umburana de cheiro/ Cerrado and Caatinga	Bark/ethanol	25 and 50 mg/kg amburoside A (Figure 4)	In vivo, rats; CCl_4_	The treatment avoided the increase in plasma AST and ALT and attenuated necrosis and infiltration of inflammatory cells in the liver. Oxidative stress was attenuated with a diminution of lipid peroxidation, restoration of the catalase activity, and reversion of the diminution in the reduced glutathione levels.	[38]
*Anacardium occidentale* (Figure 3E; Anacardia-ceae)/cashew/ Amazon rainforest, Cerrado and Caatinga	Leaves/methanol	500 and 1000 mg extract/kg, 35.5% in total phenolics (e.g., glycosylated quercetin, amentoflavone derivative)	In vivo, rats; CCl_4_	The treatment preserved the liver histo-architecture and significantly reduced the serum AST, ALT, and ALP activities.	[40,41]
*Annona crassiflora* (Anonnaceae)/araticum/ Cerrado	Bark and seeds/ethanol	Extract with 50 mg equivalents of gallic acid/kg (quercetin and rutin (Figure 4) and several organic acids)	In vivo, rats; CCl_4_	The treatment prevented lipid peroxidation, the increase in GSH, and the decrease in CAT activity. However, it did not affect significantly the changes induced by CCl_4_ on cytochrome P_450_, b_5,_ and SOD.	[39,42]
*Annona crassiflora* (Anonnaceae) araticum/ Cerrado	Fruit peel/ethanol	10–100 mg ethanolic extract/kg and 10–100 mg polyphenol-rich extract/kg	In vivo, mice. Triton WR-1339- induced hyperlipidemic mice	Lipid-lowering actions and hepatoprotective activities were found. The poly-phenols-rich fraction showed markedly stronger effects than the crude extract, with emphasis on the reduction of lipid peroxidation and protein carbonylation, in addition to the increase in thiol content and restoration of G6PD, GSH-Px, GSH-R, and GSH in the liver.	[43]
*Annona crassiflora* (Annonaceae)/araticum/ Cerrado	Fruit peel/ethanol → *n*-butanol	25, 50, and 100 mg polyphenol-rich extract per kg (procyanidin B2, epicatechin, catechin, chlorogenic acid, and caffeoyl-glucoside)	In vivo, rats; diabetes-induced oxidative and nitrosative stress	Treatment decreased serum ALT, AST, and ALP as well as hepatic lipid peroxidation, protein carbonylation and nitration, and inducible nitric oxide synthase level. The antioxidant capacity was increased as well as the glutathione reductase activity and the reduced glutathione level. A general preservation of the liver histoarchitecture was observed.	[44]
*Baccharis trimera* (Asteraceae)/gorse/ Atlantic forest	Aerial parts/hydroethanolic mixture	600 mg extract rich in quercetin (Figure 4) and flavone derivatives per kg	In vivo, rats; paracetamol	The treatment attenuated the increases in plasma ALT and AST caused by paracetamol. In addition, the treatment in-creased the CAT activity and the total concentration of glutathione but diminished the SOD activity. Histohepathologic analysis revealed a reduction in the injury caused by paracetamol.	[45]
*Baccharis trimera* (Asteraceae)/gorse/ Atlantic forest	Aerial parts/ water → ethanol precipitation	1 mg/kg of a polysaccharide fraction containing an inulin-type polysaccharide (Figure 4)	In vivo, mice; CCl_4_	At the low dose of 1 mg/kg, the preparation significantly reduced the blood levels of ALT, AST, and ALP. It also diminished lipid peroxidation and increased both the catalase activity and the levels of reduced glutathione in the liver. Administration of the polysaccharide preparation at the very high dose of 100 mg/kg increased GSH levels in the liver of heathy mice.	[46]
*Bidens pilosa* (Asteraceae)/picão-preto/ Pampa	Aerial parts/ hydro-ethanolic mixture → ethylacetate	15 mg fraction rich in quercetin derivatives (Figure 4) per kg	In vivo, mice; CCl_4_	The treatment protected the hepatic tissue by blocking lipid peroxidation, protein carbonylation, and DNA fragmentation. In addition, the plasma antioxidant capacity was preserved and there was a reduction in the elevation of the serum transaminases and lactate de-hydrogenase.	[47]
*Campomanesia adamantium* (Myrtaceae)/gabiroba/ Cerrado and Atlantic forest	Pulp and seeds/ hydro-ethanolic mixture	800 and 1000 µg extract per mL (flavonoids mainly of the flavanone and chalcone class; 138.09 mg/g)	In vitro, mice HEPG2 cells; CCl_4_	There was protection against cytotoxicity caused by CCl_4_ exposure (cell viability) and maintenance of the morphological characteristics (general and nuclear) of the cells. The treatment also reduced the appearance of AST in the supernatant of the cellular incubation medium.	[48,49]
*Caryocar brasiliense* (Caryocaraceae)/ pequi/ Cerrado	Seeds/hand pressing and cold pressing oil extraction	3 and 6 mL of hand-made and cold-pressed almond oil (fatty acids, phenolic compounds, tocopherols, carotenoids, and phytosterols) per kg.	In vivo, rats; CCl_4_	Treated rats showed diminished plasma ALT and AST levels and reduced hepatic injury scores. The plasma serum high-density lipoprotein levels were increased. In addition, the treatment improved the antioxidant capacity as revealed by the increased hepatic glutathione peroxidase and glutathione reductase activities, as well as by the reduced circulating concentrations of leptin and inflammatory mediators.	[50]
*Cecropia glaziovii* (Urticaceae)/embaúba-vermelha	Leaves/hydro- ethanolic mixture	20 mg extract/kg extract (flavone derivatives and chlorogenic acid)	In vivo, rats; CCl_4_	The extract inhibited hepatic lipid peroxidation, diminished the serum levels of ALT and AST, and increased the activities of SOD and CAT in the liver.	[51]
*Cecropia pachystachya* (Figure 3C; Urticaceae)/embaúba	Leaves/ ethylacetate	20 mg extract/kg (chlorogenic acid, iso-orientin, and orientin)	In vivo, mice; non-alcoholic liver disease induced by hyper-caloric diet	Treatment with the extract prevented the increase in liver weight and lipid per-oxidation caused by liver disease. Serum levels of ALT and AST were not affected by the treatment. Although the treatment reduced steatosis, it remained higher than in healthy animals.	[52]
*Eugenia uniflora* (Myrtaceae)/pitangueira/red Brazilian cherry)/ Cerrado, Atlantic forest and Pampa	Leaves/ ethylacetate	200 mg/kg extract (quercetin and myricetin derivatives)	In vivo, rats; CCl_4_	The treatment prevented the elevation in the serum levels of ALT, AST, total bilirubin, total cholesterol, and triglycerides. In the liver, it prevented lipid peroxidation and restored the activity of SOD and glutathione (GSH) levels. In addition, the treatment effectively attenuated the CCl_4_-induced histopathological changes.	[20]
*Hancornia speciosa* (Figure 3F; Apocynaceae)/ mangaba/ Cerrado	Fruit/freeze-dried extract	200 mg/kg extract (chlorogenic acid (150 mg/g and rutin (120 mg/g)	In vivo, rats; paracetamol	The treatment maintained the nuclear envelope integrity of the hepatocytes against damage induced by acetaminophen. It was also effective in reducing liver function markers AST, ALT, and GGT in serum. The extract also diminished lipid peroxidation and significantly improved the oxidative status of the liver by increasing the levels of antioxidant enzymes.	[53]
*Hyptis crenata* (Figure 3D; Lamiaceae)/Cerrado	Aerial parts/ steam distillation	300 mg essential oil/kg (32.8% camphor, 18.0% 1,8-cineole, 13.4% α-pinene, 12.9% β-caryophyllene; Figure 5)	In vivo, rats; poly-microbial sepsis	The essential oil normalized serum ALP, ALT, and bilirubin levels and prevented morphological changes. Additionally, the essential oil inhibited elevation in hepatic lipid peroxidation and reduction of the glutathione peroxidase activity.	[54]
*Indigofera suffruticosa* (Fabaceae)/ All biomes	Leaves/methanol	50 mg/kg (alkaloids, flavonoids, steroids, proteins, carbohydrates, triterpenes, and indigo coumarin)	In vivo, mice; paracetamol	Histopathological and histomorphometric analyses revealed the reorganization of structural units of cells, nuclei, and sinusoidal capillaries of hepatocytes by the treatment, thus reducing the damage to liver tissue and increasing organ regeneration rate. The plasma levels of the marker enzymes ALT and AST, as well as the plasma levels of bilirubin, were also restored by the treatment.	[55]
*Luehea divaricata* (Figure 3B; Malvaceae)/whips horse/Atlantic forest	Bark/hydro-ethanolic mixture	200 mg/kg (6.6 mg/kg of a β-type (epi)catechin dimer + several catechin trimers and pentamers)	In vivo, rats; paracetamol	Liver injury in rats was substantially attenuated in animals treated with the hydro-alcoholic extract (200 mg kg^−1^ day^−1^). This was deduced from aspartate aminotransferase and alanine amino-transferase measurements in plasma as well as from the hepatic activities of catalase and superoxide dismutase. The anti-inflammatory activity of the L. divaricata extract, as evidenced by the inhibitory activity of nitric oxide production, may be partly responsible for the observed hepatoprotective effects.	[56]
*Maytenus robusta* (Celastraceae)/cafezinho-do-mato/ Atlantic forest	Aerial parts/ hydro-ethanolic mixture	100 mg/kg (13.7% in phenolic compounds)	In vivo, mice; CCl_4_	Treatment with the extract reduced the hepatic histological changes and normalized the serum ALT levels. Lipo-peroxidation was diminished and the reduced glutathione levels were augmented. The activities of the following antioxidant enzymes were increased: SOD, CAT, and glutathione-S-transferase. The myeloperoxidase activity was de-creased as well as the TNFα and inter-leukin-6 levels.	[57]
*Mikania glomerata* (Asteraceae)/guaco/ Atlantic forest	Aerial parts/ water	Inulin type fructan: 100 mg/kg Fructooligosaccharides: 10 mg/kg (see Figure 4)	In vivo, mice; CCl_4_	The pretreatment with both the inulin-type fructan and the fructooligosaccharides attenuated the elevations in the serum levels of ALT, AST, and alkaline phosphatase. Further, the same pretreatments also partially prevented lipid peroxidation, the diminution in the GSH levels, and the CAT activity.	[58]
*Paullinia cupana* (Figure 3G; Sapindaceae)/guaraná/ Amazon rainforest	Seeds/water	100, 300, and 600 mg/kg extracts (vitamin C, methylxantines, catechin and epi-catechin derivatives, and pro-anthocyanidins)	In vivo, rats; CCl_4_	The guarana extracts diminished the increased serum activities of ALT and AST and prevented DNA strand breakage.	[59,60]
*Rourea induta* (Connaraceae)/chapeudinha/ Cerrado	Leaves/hydro- ethanolic mixture	500 mg extract/kg (derivatives of quercetin and hyperin)	In vivo, rats; CCl_4_	The treatment reduced the elevated serum markers ALT, AST, and bilirubin, and improved the parameters CAT, SOD, GPx, GSH, and TBARS in the hepatic tissue. Histopathologic modifications were prevented.	[61]
*Solanum fastigiatum* (Solanaceae)/falsa jurubeba/ Atlantic forest	Leaves/water	100 and 200 mg extract/kg	In vivo, mice; paracetamol	The treatment prevented the changes in the TBARS levels and non-protein thiol levels.	[62]
*Solanum paniculatum* (Figure 3H; Solanaceae)/ All biomes	Leaves/water → ethylacetate	Aqueous extract: 600–1200 mg/kg; Ethyl-acetate fraction: 300 mg/kg	In vivo, mice; paracetamol	Both the aqueous extract and its ethyl acetate fraction antagonized the rises in serum ALT and AST, though at different doses. In the liver, the same preparations antagonized the drop in reduced GSH and the increase in lipid peroxidation. The liver protective effects of the ethyl-acetate fraction were similar to those of N-acetyl-cysteine.	[63]
*Solanum paniculatum* (Figure 3H; Solanaceae)/ All biomes	Roots/hydro-methanolic mixture → ethylacetate	100 mg crude extract/kg; 200 mg 3-amino-spirostane alkaloids (jurubine, etc.) fraction per kg (Figure 5)	In vivo, mice; CCl_4_	In the groups treated with both the crude extract and the alkaloid fraction hepatic degeneration was diminished, although cytoplasmic vacuolization was still evident. Serum ALT activity was diminished, but the AST activity was not affected.	[64]
*Verbena litoralis* and *Verbena* *Montevidensis* (Verbenaceae)/fel-da-terra/ Atlantic forest	Aerial parts/water and methanol	Aqueous and methanolic extracts: 0.1–100 µg/mL Brasoside: 10–100 µM (Figure 4)	In vitro, mice HEPG2 cells; ethanol-induced injury	Toxicity was evaluated by measuring the cellular dehydrogenase activity and neutral red dye incorporation. Both aqueous and methanolic extracts of both species as well as brasoside were hepato-protective. The effects were attributed to brasoside, present in both extracts.	[65]
*Vernonia condensata* (Asteraceae)/alumã, figatil or necroton	Leaves/ethanol → ethylacetate	50–200 mg ethyl-acetate fraction per kg (rich in 1,5-di-*O*-caffeoylquinic acid; Figure 4)	In vivo, rats; paracetamol	All doses of the ethylacetate fraction reduced and eventually normalized the serum AST, ALT, and ALP levels. Moreover, all doses were able to inhibit malonaldehyde formation and increase the GSH levels. Treated rats did not present degenerative changes maintaining an almost normal histological aspect.	[66]

Abbreviations: AST, aspartate aminotransferase; ALT, alanine aminotransferase; TBARS, thiobarbituric acid reactive substances; ALP, alkaline phosphatase; GSH, reduced glutathione; CAT, catalase; SOD, superoxide dismutase; HDL, high-density lipoproteins GPx, glutathione peroxidase.

The use of leaves of fruit trees as byproducts is remarkable as a form of sustainable production if one considers the possibility of obtaining various different products from the same plant. The leaves of the cashew tree (*Anacardium occidentale),* Surinam cherry (*Eugenia uniflora),* and jurubeba (*Solanum fastigiatum*) are good examples of perspective value aggregation to unusual plant parts as sources of potential bioactives. In the same way, several other non-fruit species that are quite common in the Brazilian flora, such as *Cecropia glaziovii* (embaúba-vermelha, Ambay pumpwood), may have their bioactive potential explored. Sustainable exploration of native plants of the Brazilian biomes is also interesting thanks to the easiness of both planting and cultivation. Additionally, this kind of exploration is also important for protecting the species from degradation and extinction.

The hepatoprotective effectiveness of the plant preparations is an important issue because it largely determines the doses that have to be ingested by the eventual future patients. As is always the case with therapeutic drugs in general, the lowest possible doses are highly desirable for several reasons especially because this, in principle, also minimizes undesired side effects. Whenever possible, the active doses utilized in the studies were given in Table 1. They vary largely, actually between 1 and 1000 mg per kg. All in vivo experiments, however, were done with rats or mice, so translation to humans is recommended. The translation formulas presented by Reagan et al. [67] can be used at least for obtaining preliminary insight. For translating the data obtained with rats the formula is: possible human dose (mg/kg) = active dose in rats × 0.161; for mice, it would be: possible human dose (mg/kg) = active dose in mice × 0.081. The *Anacardium occidentale* (cashew) active leave extract doses in rats were 500 and 1000 mg/kg [40].

Applying the above formula, the active doses in humans can be expected to be 80.5 and 161.0 mg/kg. For an individual weighing 75 kg, this means that doses of 6.0 and 12.0 g would be necessary. These are high doses, but the oil of *Caryocar brasiliense* seeds (pequi) shows an even less favorable perspective [50]. The 3 or 6 mL per kg in rats would mean 36 and 72.5 mL for an individual weighing 75 kg.

Evidently, such high doses of preparation are not very realistic from both therapeutic and behavioral perspectives. Daily doses of at least as low as 1 g per day would be highly desirable. This is achieved by preparations requiring 82.7 mg/kg in rats and 164.6 mg/kg in mice (for an individual of 75 kg). Clinical studies with silymarin preparations, the classical hepatoprotective agent of plant origin (*Silybum marianum*), for example, have used doses between 135 and 600 mg/day [4,5]. On the other hand, there are several preparations listed in Table 1 that fulfill this criterium. The *Bidens pilosa* extract is a very good example [47]: the 15 mg/kg dose in mice would be equal to 91 mg for an individual of 75 kg. Similar extracts that would require low doses are those from *Cecropia glaziovii* [51] and *Cecropia pachystachya* [52]. These preparations doses of 241.5 and 121.5 mg, respectively, can be expected to be active in an individual weighing 75 kg. The most effective preparation listed in Table 1, however, was prepared from *Baccharis rimera* (gorse) [45]: applying the same calculation one arrives at a putative human dose of 81 µg/kg, or 6 mg for a 75 kg weighing individual. It is true that in this case, the preparation is not a crude extract, as it represents in fact a semi-purified polysaccharide fraction containing an inulin-type polysaccharide. Even so, it could prove to represent a viable and promising preparation if its effectiveness in humans turns out to be similar to that one reported for mice. Perhaps it is worth adding some comments about the difference between crude or relatively crude extracts and purified or semi-purified compounds. A dose of 500 or even 1000 mg of an extract, if its effectiveness is proven, may be viable in economic terms, whereas much lower doses of purified or semi-purified compounds may not. For example, amburoside A, extracted from *Amburana cearenses* [38], was hepatoprotective in rats at doses of 25 and 50 mg/kg. Translation to humans would lead to doses of 302 and 604 mg, respectively. These doses fulfill the criterium stipulated above of a dose at least under 1000 mg for an adult individual, but the economic viability of preparing the large amounts of pure amburoside A that are required is a crucial question. The costs can be impeditive, and, in many cases, it is likely that crude extracts will be preferable if one takes into account the economic constraints.

### 5.3. The Folk Medicinal Perception of Brazilian Hepatoprotective Plants

A question that might be formulated is one about the popular perception of the possible hepatoprotective action of the plants listed in Table 1. Among all the 21 species listed in Table 1, for just eight there are in fact popular manifestations that they are ingested for hepatoprotective purposes. These species are: *Baccharus trimera* [45,46], *Hyptis crenata* [54], *Indigofera suffruticosa* [55], *Solanum fastigiatum* [62], *Solanum paniculatum* [63,64], *Verbena litoralis* [65], *Verbena montevidensis* [65], and *Vernonia condensata* [66]. For several others, however, there are claims about protection against illnesses that are in a certain way related to at least one aspect that is usually present in liver injuries, such as inflammation, for example.

Some of the fruits listed in Table 1 have gained commercial notoriety in Brazil and elsewhere such as guarana (*Paullinia cupana*), pequi fruit (*Caryocar brasiliense*), and mangaba (*Hancornia speciosa*). All three species are largely consumed in various forms. The guarana fruits are used as an ingredient of a famous and widely commercialized soft drink. However, none of them is popularly mentioned as being hepatoprotective, even though that mangaba is considered anti-inflammatory [53]. This has a relation, perhaps, with the fact that relatively high doses are most likely to be needed in humans for significant hepatoprotective action. On the other side of the spectrum, *Verbena litoralis* and *Verbena montevidensis* (fel-da-terra), are species for which a strong popular consensus exists about their putative hepatoprotective effects [65]. Unfortunately, the study consisted of in vitro experiments, so there is no means of inferring about the doses that are effectively needed in rodents or humans.

## 6. Compounds Possibly Involved in Hepatoprotection and Molecular Mechanisms

**Figure 4:**
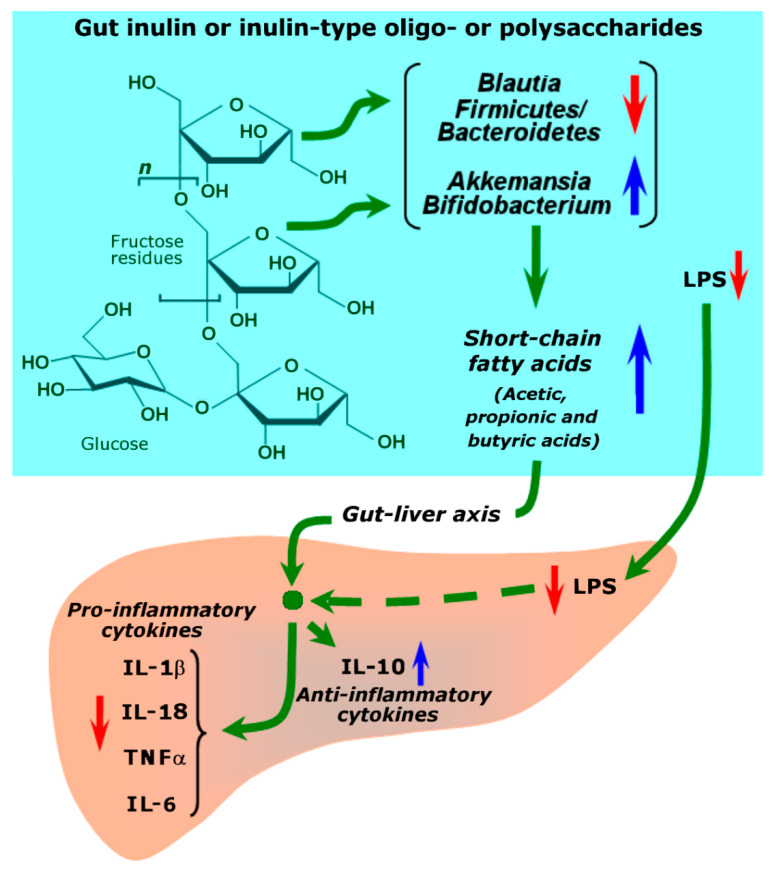
Possible mechanism of hepatoprotection by inulin polysaccharide or inulin oligosaccharides through the gut-liver axis. LPS refers to the gut microbial lipo-polysaccharide.

**Figure 5:**
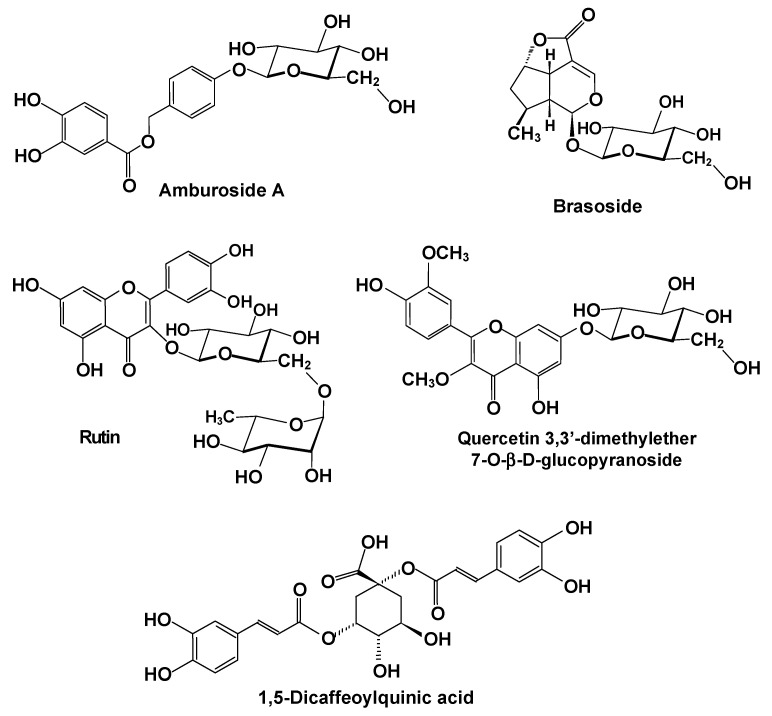
Brasoside and examples of phenolic compounds possibly involved in the hepatoprotective action of several Brazilian plants.

**Figure 6:**
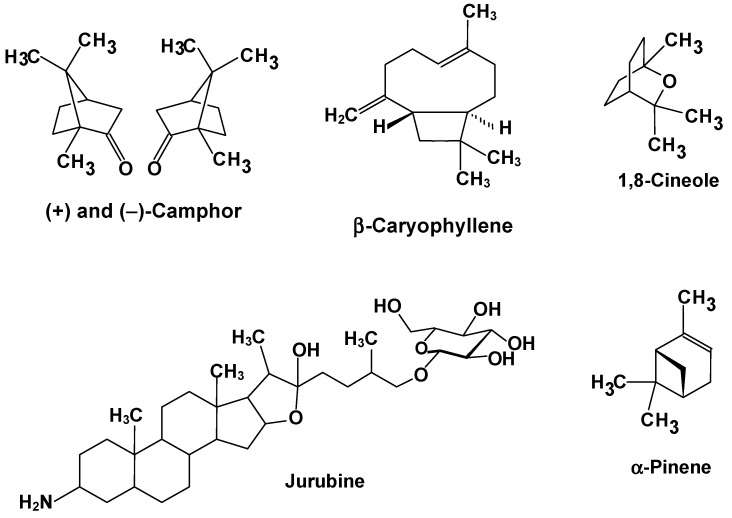
Examples of lipophilic compounds and the saponin jurubine, possibly involved in the hepatoprotective action of several Brazilian plants.

Table 1 also informs about the bioactive compounds that are possibly involved in the reported effects. Representative structures are shown in Figure 4, Figure 5 and Figure 6. Figure 4 combines the structural features of the inulin-type fructans with a representation of their possible mode of action. Figure 5 shows several representative structures of bioactives that generally bear polyphenolic structures and that can be regarded as more or less water soluble. Compounds of this group are quite frequently mentioned in the studies listed in Table 1. Figure 6, finally, shows representative structures of compounds that can be regarded basically as lipid-soluble ones, such as components of essential oils and alkaloids with a highly lipophilic core. The compounds represented in Figure 4, Figure 5 and Figure 6 should by no means be regarded as representing an exhaustive list of the molecular agents involved in the hepatoprotection of Brazilian plants. They should merely be regarded as a representative selection of typical bioactive structures involved in hepatoprotection.

Establishing the compound or group of compounds responsible for the hepatoprotective actions is important if the aim is to determine the mechanism underlying these phenomena. Most of the studies listed in Table 1 were accompanied by some effort at identifying the compound or compounds involved. In some cases, specific compounds were indicated as the most probable agents; in most cases, however, it was speculated about the participation of a more or less ample group of frequently related substances. As mentioned above, the hepatotoxic agents are precursors of highly reactive molecules, potentially harmful to organic structures which are composed of proteins, lipids, and nucleic acids. Additionally, the inflammation that derives from the damage is able to induce and amplify oxidative stress in the tissue. Hepatoprotective agents must thus be capable of neutralizing these deleterious actions. If one examines the mechanisms that have been proposed for hepatoprotection by natural compounds, two main modes of action can be highlighted for the compounds that are mentioned in the 26 studies listed in Table 1. These two main modes are: (a) an indirect action using the gut-liver axis; (b) a direct action that results from the anti-inflammatory and antioxidant properties of the compound. The indirect action does not require direct access of the compound to the liver via circulation. It should also be mentioned beforehand that a direct action of a given compound, or group of compounds, does not exclude a simultaneous action via the gut-liver axis.

An indirect action via the gut-liver axis is likely to occur in the preparations of *Baccharis trimera* [45] and *Mikania glomerata* [58], which contain inulin-type poly- and oligosaccharides. Inulin is a heterogeneous group of fructose polymers formed by repetitive fructosyl residues with a terminal glucosyl moiety (see Figure 4). The fructosyl residues are linked by β(2,1) bonds [68]. The β(2,1) linkages are not digested by the human and rodent alimentary systems. However, fructose poly- and oligosaccharides (up to ten residues) may act via regulation of the gut microbial community. There are several studies showing that inulin attenuates metabolic disorders by improving serum lipid, fasting blood glucose, and insulin resistance by restoring or changing the gut microbiota composition [69,70]. The gut microbiota produces several signaling molecules such as LPS (lipopolysaccharide) and short-chain fatty acids, which include acetic, propionic, and butyric acids. LPS is a bacterial endotoxin, derived from intestinal Gram-negative bacteria. Normally, only small amounts of LPS translocate through the gut and reach the liver, where it is taken up by Kupffer cells and removed. This action prevents the activation of the toll-like receptor 4 (TLR4), which promotes the release of inflammatory cytokines (see Figure 4). Failure in lowering this activation process, which may occur during liver disease, further promotes liver damage via the action of inflammatory cytokines. It has been shown that ingestion of inulin by mice with non-alcoholic fatty liver disease significantly reduces the LPS levels in both serum and liver tissue and the expression of the toll-like receptor 4 [70]. Furthermore, treatment with inulin reduced the production of short-chain fat in the intestine. The latter is probably the consequence of modifications in the gut microbiota. For instance, Bao et al. [70] found that the abundance of *Akkermansia* and *Bifidobacterium* exhibited positive correlations with the production of short-chain fatty acids and IL-10. IL-10 is an anti-inflammatory cytokine, whereas short-chain fatty acids trigger the system that downregulates the production of inflammatory cytokines (Figure 4) and up-regulates the production of IL-10. Opposed to these observations, the abundance of *Blautia* in the gut, for example, was negatively correlated with short-chain fatty acid production. These and other observations strongly suggest that the actions of the fructan-rich *Baccharis trimera* [45] and *Mikania glomerata* [58] extracts against the hepatic disease occur via the gut-liver axis, as illustrated by Figure 4.

The classical example of a preparation of plant origin that acts, predominantly at least, directly on the hepatic tissue is the mixture of polyphenolic compounds known as silymarin [4,71]. Silymarin, which exerts antioxidant, immunomodulatory, and antifibrotic activities, is able to affect the synthesis of RNA and DNA and maintains the integrity of the hepatocyte membrane. In several of the studies listed in Table 1, silymarin was used as a positive control. Up to now, none of the silymarin components have been reported as a constituent of the 22 Brazilian plant species listed in Table 1. Polyphenolic compounds, such as flavonoids and phenolic acids, however, have been quite prominent in most studies on Brazilian plants with hepatoprotective activity, as shown in Table 1. Figure 4 illustrates the kind of structures involved. Most of the flavonoids and phenolic acids present in the plant extracts are linked to carbohydrate moieties, for example, the quercetin derivative shown in Figure 5, rutin, and amburoside A. In other cases, they appear in condensed forms as dimers, trimers, etc., as is the case of 1,5-dicaffeoylquinic acid. There is ample experimental evidence that quercetin or quercetin derivatives and rutin are able to protect the liver against diseases and damage caused by hepatotoxins [72,73,74]. This seems also to be valid for amburoside A, which is quite abundant in the *Amburana cearenses* extract [38]. Polyphenolic compounds are no doubt highly effective antioxidants in general, a property that might explain their effects against liver damage injury in which oxidative stress plays a highly significant role. The classical hepatoprotective agent silibinin, alone or in combination with other analogs, apparently acts directly against oxidative stress and sustained inflammatory processes [4,5].

On the other hand, for exerting such an effect their concentrations in the hepatic tissue must be appropriate. This was certainly the case in those experiments listed in Table 1, where isolated cells were used. In the experiments in which the hepatoprotective action of *Campomanesia adamantium* extract was investigated [48], for instance, the cells were exposed to a total polyphenolic concentration from 110.4 to 138.1 µg/mL. Such relatively high concentrations are not normally reported for systemic circulation after oral ingestion. Actually, concentrations above 10 µmol/L are not very easy to achieve, with concentrations under 1 µmol/L being a frequent occurrence [75,76]. However, blood is mostly carried to the liver via the portal vein, in which the concentrations of polyphenolics that reach the liver are certainly much higher than those in systemic circulation. Even so, it can be hypothesized with some confidence that the concentration of the polyphenolics that reach the liver is in general relatively modest and that only compounds that are fairly strong antioxidant agents may in fact be acting as antioxidants and anti-inflammatories in a direct way. This is the reason why other mechanisms, which are not necessarily linked to direct antioxidant action, have been suggested and also investigated for natural polyphenolics. The flavonoid naringenin has been the object of such studies [19]. This compound alone presented most of the effects that were reported by the studies listed in Table 1 against liver injury induced by CCl_4_. In addition, it was found that naringenin attenuated liver inflammation by downregulating the CCl_4_-induced activation of tumor necrosis factor-alpha (TNF-α), inducible nitric oxide synthase (iNOS), and cyclo-oxygenase (COX-2) at both the protein and mRNA levels. This might have been a direct effect on the hepatic tissue, but the gut-liver axis may also have been involved. This is suggested by another study with mice bearing non-alcoholic fatty liver disease in which it is shown that naringin (the aglycone of naringenin) modifies the community compositional structure of the gut bacteria characterized by increased benefits and fewer harmful bacteria [77]. Similar results were obtained with ellagic acid, a phenolic acid found in many plants, that also acts as a hepatoprotective agent [78]. Administration of this compound to mice with alcoholic liver disease also significantly improved the microbiota dysbacteriosis [79]. Ellagic acid also appears frequently in several plants in the form of glycosylated derivates, usually named ellagitannins. There are several reports about the antioxidant, anti-inflammatory, and, especially, hepatoprotective effects of this class of compounds, sometimes with an effectiveness comparable to that of silymarin [80,81]. All these results clearly suggest that the action of polyphenolics on liver diseases is not solely the result of their direct action as antioxidants or anti-inflammatories, but also the consequence of their activities as down- and up-regulators of various signaling pathways and that participation of the liver-gut axis is equally possible.

Figure 6 presents another group of substances possibly involved in the hepatoprotective effects listed in Table 1, whose nature differs substantially from those in Figure 5. Most of the compounds in Figure 5 are strongly lipophilic, frequent components of essential oils such as those extracted from *Hyptis crenata* [54] and *Caryocar brasiliense* [50]. Jurubine, on the other hand, a constituent of *Solanum paniculatum* roots [64,82], is a nitrogenous saponin, in which the lipophilic aglycone structure is balanced by polar groups at both ends of the molecule [83]. The *Hyptis crenata* study provides an interesting example because the essential oil fraction consists of 77.1% of four compounds, namely camphor, 1,8-cineole, α-pinene, and β-caryophyllene [54]. These are common components of essential oils that have also been found to possess hepatoprotective properties. A recent review mentions 27 species whose essential oils have been reported to possess hepatoprotective activity [84]. Some of their molecular components have been tested in pure form. A good example is β-caryophyllene, whose action in mice with nonalcoholic steatohepatitis was investigated [85]. Beta-caryophyllene attenuated many modifications caused by the disease. It attenuated, for example, the mRNA expression of the monocyte chemotactic and activating factor of fibrosis-related factors and of antioxidant-related factors [85]. The antioxidant action of β-caryophyllene in vivo was confirmed by another study with arthritic rats, in which the liver also undergoes substantial damage [86]. In these animals, β-caryophyllene abolished the increases of protein carbonyl groups and myeloperoxidase activity in the liver and plasma and restored the increased levels of reactive oxygen species, and reduced glutathione.

## 7. Conclusions and Perspectives

The information that was collected in the literature indicates that a fair number of Brazilian native plants with folk medicinal use present hepatoprotective properties. These properties can be attributed to various compounds such as poly- and oligosaccharides, polyphenolics and derivatives, and components of essential oils. This reinforces the widespread notion that plants, sources of bioactive compounds, can be regarded as an alternative for treating hepatic illnesses.

The study of native plants with hepatoprotective potential is crucial for the discovery of new compounds that can be used at least as adjuvants against hepatic illnesses. However, the number of Brazilian plants that have been investigated in this respect is still relatively modest if one considers the richness of the Brazilian flora and more detailed experimental evidence is still lacking. Experimental evidence is especially scarce in terms of inflammatory mediators and gene expression. On the other hand, apparently, no detailed and comprehensive clinical studies and trials were conducted up to now using Brazilian native plants aiming specifically at a potential hepatoprotective effect, as has been done with silymarin, for example [4,5]. Consequently, one is forced to conclude that the subject analyzed in this review is still in the pre-clinical stage.

On the other hand, the extracts discussed herein are remarkable because they were mostly obtained from parts with low commercial potential, such as barks, seeds, and leaves from fruit trees. This is a feature that could eventually contribute substantially to economic development and sustainable production because almost no additional productive potential and infrastructure are needed. In addition, it could represent a possibility of increasing revenue due to value aggregation to otherwise low-valued products.

## Data Availability

Not applicable.
